# Venous Thromboembolism in Aggressive B‐Cell Lymphoma Patients Treated with CD19 CAR‐T Therapy: Single‐Institution Study

**DOI:** 10.1002/jha2.70227

**Published:** 2026-01-30

**Authors:** Neha Venkatesh, Kevin Milligan, Julia Parrish, Yun Qing, Ryan Sun, Paolo Strati, Jeremy Ramdial, Amy Ayers, Sairah Ahmed, Cristhiam Rojas Hernandez

**Affiliations:** ^1^ Baylor College of Medicine Houston Texas USA; ^2^ Department of Biostatistics The University of Texas MD Anderson Cancer Center Houston Texas USA; ^3^ Department of Lymphoma and Myeloma The University of Texas MD Anderson Cancer Center Houston Texas USA; ^4^ Department of Stem Cell Transplantation Division of Cancer Medicine The University of Texas MD Anderson Cancer Center Houston Texas USA; ^5^ Section of Benign Hematology The University of Texas MD Anderson Cancer Center Houston Texas USA

## Abstract

**Background:**

Large B‐cell lymphomas (LBCLs) are a common subtype of non‐Hodgkin lymphomas. CD19 chimeric antigen receptor T‐cell (CAR‐T) therapy has revolutionized LBCL treatment, with high remission rates but also significant toxicities, including cytokine release syndrome (CRS) and immune effector cell‐associated neurotoxicity syndrome (ICANS). Venous thromboembolism (VTE) in CAR‐T therapy patients is understudied.

**Objective:**

This study aims to determine the incidence and characteristics of acute VTE in LBCL patients treated with CAR‐T therapy and identify baseline clinical features associated with VTE. Methods: We retrospectively reviewed 172 adult LBCL patients treated with CAR‐T therapy from January 2018 to November 2019 at MD Anderson Cancer Center. Data on demographics, clinical characteristics, and adverse events were collected. VTE events within 6 months post‐CAR‐T therapy were confirmed by diagnostic imaging. Statistical analyses included univariate analyses and cumulative incidence functions.

**Results:**

The cohort was predominantly male (70%), with a median age of 59 years and advanced‐stage disease (76.16%). The 6‐month incidence of VTE was 7.6%, primarily involving upper extremity events related to central venous catheters. Significant associations were found between VTE and disease histology (*p* = 0.033) and high‐grade ICANS (*p* = 0.013). PMBCL patients had a higher VTE incidence (30%) compared to DLBCL and TFL. Most VTE events occurred within the first month post‐CAR‐T therapy.

**Conclusion:**

LBCL patients receiving CAR‐T therapy have a significant risk of VTE, particularly within the first month and among those with PMBCL and high‐grade ICANS. This highlights the need to study the role of venous thromboprophylaxis in this context.

## 1 Introduction

Large B‐cell lymphomas (LBCLs) are a common subtype of NHLs, comprising up to 40% of cases globally, with an estimated incidence of 7 per 100,000 in the United States [1, 2].

CD19 chimeric antigen receptor T‐cell (CAR‐T) therapy has revolutionized LBCL treatment, with studies reporting complete remission rates as high as 63.6% in a refractory disease setting [3]. CAR‐T cells target specific tumor antigens, effectively killing tumor cells expressing the CD19 antigen, which is present in 95% of LBCL cases [3]. Despite its success, CAR‐T therapy comes with a unique toxicity profile, including most commonly cytokine release syndrome (CRS) and with neurologic toxicity termed immune effector cell‐associated neurotoxicity syndrome (ICANS). CRS can lead to multi‐organ toxicities, including hematological complications [4]. Among hematological complications, venous thromboembolism (VTE) has not yet been characterized in recipients of CAR‐T.

Although VTE is a significant cause of morbidity and mortality in hematological malignancies, data on thrombotic complications in patients receiving CAR‐T therapy are limited. Preliminary evidence suggests that CAR‐T therapy can cause immune‐mediated endothelial activation and suppressed fibrinolysis, potentially leading to thrombosis [5]. Patients receiving CAR‐T therapy often have advanced‐stage disease and undergo invasive procedures, further increasing their thrombosis risk [6]. In addition, high levels of interleukin‐6 (IL‐6) and specific genetic polymorphisms such as the IL‐6 promoter polymorphism (−57 C/G) seen in patients receiving CAR‐T therapy may contribute to this risk [7].

Existing studies indicate that VTE in CAR‐T therapy patients can manifest as pulmonary embolism, or upper or lower extremity deep vein thrombosis [8]. Risk factors include a history of recent VTE, a body mass index (BMI) ≥ 30, recent procedures like central venous catheter (CVC) insertion (especially in upper extremities), intensive care unit (ICU) stays, and recent infections [8, 9]. Managing anticoagulation is challenging due to post‐therapy thrombocytopenia [5, 8].

Given the limited data on VTE in CAR‐T therapy patients, our study aims to determine the incidence and characteristics of acute VTE in LBCL patients treated with CAR‐T therapy, and to identify baseline clinical features associated with VTE.

## 2 Methods

### 2.1 Study Design and Population

The study included consecutive adult patients who had ever received autologous anti‐CD19 axicabtagene ciloleucel CAR‐T therapy for aggressive B‐cell lymphoma at our institution from January 1, 2018, to November 9, 2019.

Patients were identified through an institutional database and the study was approved by The University of Texas MD Anderson Cancer Center Institutional Review Board and was conducted in accordance with the institutional guidelines and principles of the Declaration of Helsinki. The clinical characteristics and laboratory features were collected before the initiation of lymphodepleting chemotherapy.

Demographic and clinical characteristics were collected at the time of CAR‐T cell therapy. CRS and ICANS were prospectively graded and managed according to the ASTCT guidelines [10]. Response status was determined by the Lugano 2014 classification. Incidence and date of relevant adverse events, such as infections and cytopenia, were collected. VTE‐related data, such as history of VTE, presence of CVCs, and other comorbidities using the Carlson index, were collected within the following 6 months from CAR‐T.

### 2.2 Outcome Definition

Individual medical records of identified patients were manually screened for acute VTE complications within 6 months of CAR‐T cell therapy infusion. All events were confirmed by diagnostic imaging modalities.

### 2.3 Statistical Analysis

Summary statistics were provided for patient demographics as well as clinical and biological variables. Continuous variables were presented as mean ± SD or median (interquartile range [IQR]), as appropriate based on normality, and categorical variables were presented as percentages. Continuous data were compared using the unpaired Student's *t*‐tests or Wilcoxon rank‐sum tests, as appropriate. Categorical data was compared using the Fisher's exact test. Univariate analyses were performed. Statistical significance was defined using a 2‐tailed *p* value ≤ 0.05. To quantify the risk of VTE, we used the cumulative incidence function. To compare cumulative incidence functions of VTE and death, we use Gray's test.

## 3 Results

### 3.1 Baseline Characteristics

A total of 172 patients were included in the study. Patients were predominantly male (70%) with a median age of 59 years. Tumor histology categories were diffuse large B cell lymphoma (DLBCL), primary mediastinal large B cell lymphoma (PMBCL), or transformed follicular lymphoma (TFL), and 76.16% had Stage III or IV disease. Patients had received more than 2 lines of cancer therapy (79.07%) before receiving CAR‐T cell treatment. Most patients had a performance status of ECOG of 0 or 1 (88.37%).

A minority of patients had prior history of VTE (29.65%), prior anticoagulation use (32.56%), or prior antiplatelet use (26.74%). All patients were on pharmacological VTE prophylaxis unless there was a bleeding contraindication or platelet counts were < 50 K/µL, at which time for patients without prior VTE, prophylaxis would be held. For patients who were on anticoagulation for a previous thrombosis, dosing would be therapeutic and held when platelet counts were < 50/µL. We did not reliably capture the frequency and compliance use of mechanical VTE prophylaxis (i.e., pneumatic compression devices) when pharmacological VTE prophylaxis was contraindicated. Patients were not routinely prescribed outpatient VTE prophylaxis after hospital discharge from CART infusion.

After CAR‐T cell treatment, a small subset of patients had severe toxicities such as ICANS Grade 3 or 4 (35.47%) or CRS Grade 3 or higher (7.56%) from CAR‐T cell therapy (Table 1).

**TABLE 1:** Demographics and clinical characteristics of 172 lymphoma patients who underwent CAR‐T therapy.

Characteristic and categories	*N* (%)
Age (years)	
Median, (IQR)	59, (49–67)
Sex	
Female	51 (29.65)
Male	121 (70.35)
Diagnosis	
DLBCL	133 (77.33)
PMBCL	10 (5.81)
TFL	29 (16.86)
Stage	
I	4 (2.33)
I/II	16 (9.30)
II	11 (6.40)
III	10 (5.81)
III/IV	63 (36.63)
IV	68 (39.53)
ECOG	
0	49 (28.49)
1	103 (59.88)
2	12 (6.98)
3	7 (4.07)
4	1 (0.58)
IPI score	
0	6 (3.49)
1	21 (12.21)
2	49 (28.49)
3	50 (29.07)
4	44 (25.58)
5	2 (1.16)
Previous therapies > 2	
No	36 (20.93)
Yes	136 (79.07)
Received bridging therapy	
No	85 (49.42)
Yes	87 (50.58)
Received bridging chemotherapy	
No	119 (69.19)
Yes	53 (30.81)
Received autologous HSCT	
No	127 (73.84)
Yes	45 (26.16)
Received allogeneic HSCT	
No	169 (98.26)
Yes	3 (1.74)
Baseline ferritin without and with DVT	
No	1704 (1364)
Yes	890 (1373)
Prior history of VTE	
No	121 (70.35)
Yes	51 (29.65)
Prior anticoagulation use	
No	116 (67.44)
Yes	56 (32.56)
Prior antiplatelet use	
No	126 (73.26)
Yes	46 (26.74)
ICANS Grade 3 or 4	
No	111 (64.53)
Yes	61 (35.47)
CRS Grade 3 or higher	
No	159 (92.44)
Yes	13 (7.56)

Abbreviations: CART, chimeric antigen receptor T‐cell; CRS, cytokine release syndrome; DLBCL, diffuse large B cell lymphoma; ECOG, Eastern Cooperative Oncology Group; HSCT, hematopoietic stem cell transplant; ICANS: immune effector cell‐associated neurotoxicity syndrome; IPI: International Prognostic Index; PMBCL, primary mediastinal B cell lymphoma; TFL, transformed follicular lymphoma.

### 3.2 Venous Thromboembolic Complications

**FIGURE 1:**
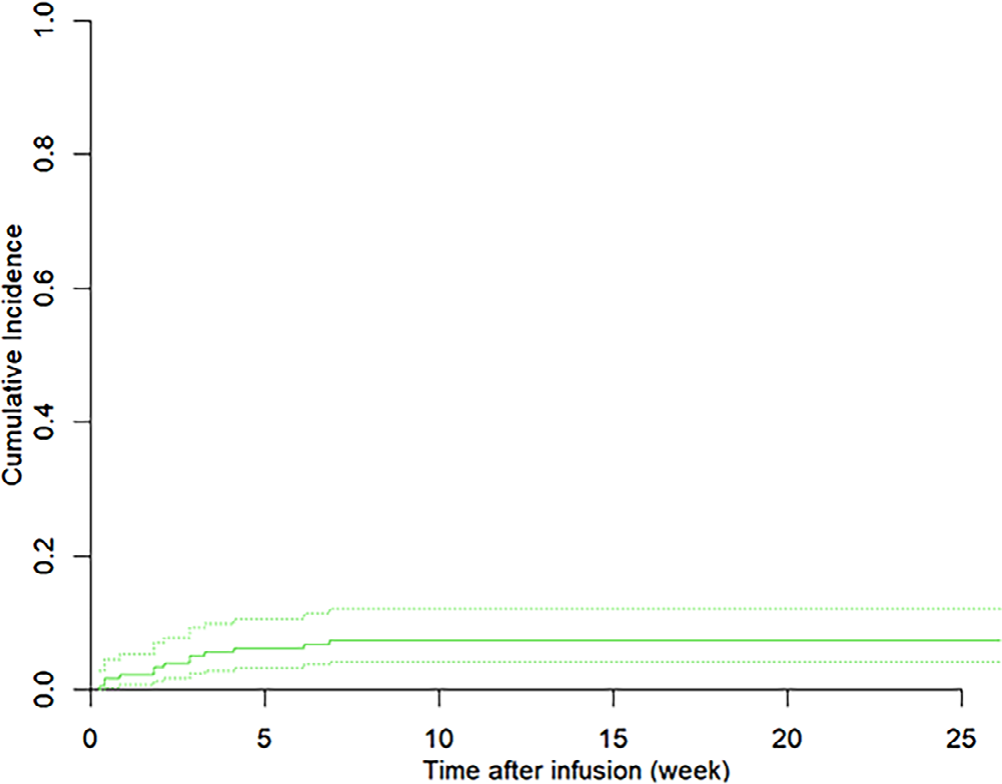
Time to VTE counting death as competing risk.

The incidence of deep VTE at 6 months from CAR‐T cell therapy was 7.6%, and the cumulative incidence estimate, counting death as a competing risk estimate was similar (Figure 1). Out of those events, 10/13 events were in the upper extremity (UE) and related to CVCs. Of these, 12/13 events were proximal DVTs in the axillary or femoral veins. In addition, 11/13 patients with DVT had peripherally inserted central catheters (PICC). Seven of the UE events were symptomatic, while two events were found incidentally in other imaging modalities. The rest of VTE events were occurred in lower extremities and were all symptomatic. No events of pulmonary embolism were observed. No fatality was observed due to VTE.

Univariate analysis for demographics, clinical characteristics, and VTE development showed a significant relationship between histology of disease (*p* value 0.033), and high‐grade ICANS (grade ≥ 3) (*p* value 0.013). Patients with PMBCL developed VTE (30%, *n* = 3) almost five times as much as patients with DLBCL (6.77%, *n* = 9) and almost 10 times more than patients with TFL (3.45%, *n* = 1) (Table 2).

**TABLE 2:** Univariate analysis for demographics, clinical characteristics, and VTE development in lymphoma patients receiving CAR‐T therapy.

Characteristic and categories	No VTE (*n* = 159), *n* (%)	Developed VTE (*n* = 13), *n* (%)	Total (*n* = 172), *n* (%)	*p* value
Age				0.572
Under 60	88 (93.62)	6 (6.38)	94 (100.00)	
Over 60	71 (91.03)	7 (8.97)	78 (100.00)	
Sex				1
Female	47 (92.16)	4 (7.84)	51 (100.00)	
Male	112 (92.56)	9 (7.44)	121 (100.00)	
Diagnosis				0.033
DLBCL	124 (93.23)	9 (6.77)	133 (100.00)	
PMBCL	7 (70.00)	3 (30.00)	10 (100.00)	
TFL	28 (96.55)	1 (3.45)	29 (100.00)	
Stage				0.737
I	4 (100.00)	0 (0.00)	4 (100.00)	
I/II	14 (87.50)	2 (12.50)	16 (100.00)	
II	11 (100.00)	0 (0.00)	11 (100.00)	
III	9 (90.00)	1 (10.00)	10 (100.00)	
III/IV	57 (90.48)	6 (9.52)	63 (100.00)	
IV	64 (94.12)	4 (5.88)	68 (100.00)	
ECOG				0.395
0	47 (95.92)	2 (4.08)	49 (100.00)	
1	94 (91.26)	9 (8.74)	103 (100.00)	
2	10 (83.33)	2 (16.67)	12 (100.00)	
3	7 (100.00)	0 (0.00)	7 (100.00)	
4	1 (100.00)	0 (0.00)	1 (100.00)	
IPI score				0.901
0	6 (100.00)	0 (0.00)	6 (100.00)	
1	20 (95.24)	1 (4.76)	21 (100.00)	
2	46 (93.88)	3 (6.12)	49 (100.00)	
3	46 (92.00)	4 (8.00)	50 (100.00)	
4	39 (88.64)	5 (11.36)	44 (100.00)	
5	2 (100.00)	0 (0.00)	2 (100.00)	
Previous therapies > 2				1
No	34 (94.44)	2 (5.56)	36 (100.00)	
Yes	125 (91.91)	11 (8.09)	136 (100.00)	
Received bridging therapy				0.248
No	81 (95.29)	4 (4.71)	85 (100.00)	
Yes	78 (89.66)	9 (10.34)	87 (100.00)	
Received bridging chemotherapy				1
No	110 (92.44)	9 (7.56)	119 (100.00)	
Yes	49 (92.45)	4 (7.55)	53 (100.00)	
Received autologous HSCT				1
No	117 (92.13)	10 (7.87)	127 (100.00)	
Yes	42 (93.33)	3 (6.67)	45 (100.00)	
Received Allogeneic HSCT				0.211
No	157 (92.90)	12 (7.10)	169 (100.00)	
Yes	2 (66.67)	1 (33.33)	3 (100.00)	
Prior history of VTE				0.531
No	113 (93.39)	8 (6.61)	121 (100.00)	
Yes	46 (90.20)	5 (9.80)	51 (100.00)	
Prior anticoagulation use				1
No	107 (92.24)	9 (7.76)	116 (100.00)	
Yes	52 (92.86)	4 (7.14)	56 (100.00)	
Prior antiplatelet use				1
No	116 (92.06)	10 (7.94)	126 (100.00)	
Yes	43 (93.48)	3 (6.52)	46 (100.00)	
ICANS Grade 3 or 4				0.013
No	107 (96.40)	4 (3.60)	111 (100.00)	
Yes	52 (85.25)	9 (14.75)	61 (100.00)	
CRS Grade 3 or 4				0.256
No	148 (93.08)	11 (6.92)	159 (100.00)	
Yes	11 (84.62)	2 (15.38)	13 (100.00)	

Abbreviations: CART, chimeric antigen receptor T‐cell; CRS, cytokine release syndrome; DLBCL, diffuse large B cell lymphoma; ECOG, Eastern Cooperative Oncology Group; HSCT, hematopoietic stem cell transplant; ICANS: immune effector cell‐associated neurotoxicity syndrome; IPI: International Prognostic Index; PMBCL, primary mediastinal B cell lymphoma; TFL, transformed follicular lymphoma; VTE, venous thromboembolism.

Overall survival analysis in this cohort showed a significantly reduced OS association in PMBCL patients with VTE, and if ICANS Grade 3 or 4 toxicity was observed in those with VTE (Table 3). The covariates that were controlled for are diagnosis (all, DLBCL, PMBCL, or TFL) and whether ICANS Grade 3 or 4 was present or not. Time to event analysis of VTE with death as competing risk showed that out of 172 patients, 64 patients died, and of those, 43 died without VTE while 15 died with unknown VTE status. Out of all patients who died, six patients presented with VTE within 6 months of CAR‐T therapy. For patients with PMBCL, the 6 month incidence of acute VTE was 30%, and for those who developed ICANS Grade 3 or 4, it was 15%. For all patients, 4‐week cumulative incidence of VTE was 5.81% and, 8‐week cumulative incidence of VTE was 7.56%, showing us that most thrombotic events took place in the first month of CAR‐T therapy initiation.

**TABLE 3:** Multivariate analysis of VTE in lymphoma patients undergoing CAR‐T therapy.

Variable	Categories	Number of patients	Number of VTE	OR (95% confidence limits)	*p* value
Diagnosis	All patients	172	13		
	DLBCL	133	9	Reference group	
	PMBCL	10	3	6.546 (1.169, 32.36)	0.022
	TFL	29	1	0.612 (0.032, 3.640)	0.7
ICANS Grade 3 or 4	No	111	4	Reference group	
	Yes	61	9	4.672 (1.398, 18.65)	0.017

Abbreviations: CART, chimeric antigen receptor T‐cell; DLBCL, diffuse large B cell lymphoma; ICANS, immune effector cell associated neurotoxity syndrome; PMBCL, primary mediastinal B cell lymphoma; TFL, transformed follicular lymphoma; VTE, venous thromboembolism.

## 4 Discussion

The incidence of VTE at 6 months after axicabtagene ciloleucel infusions of CAR‐T cell therapy was 7.6% in our cohort, in which all patients had central venous access. Comparatively, the incidence of thrombosis after conventional chemotherapy traditionally varies across studies due to different patient populations, types of lymphoma, chemotherapy regimens, and study designs. The overall global incidence rate of thrombosis in lymphoma patients, including both venous and arterial events, is reported at 6.4% [11]. These events are more frequent in the initial cycles of chemotherapy, particularly by the third cycle [12]. Key risk factors include the use of specific chemotherapeutic agents like doxorubicin or methotrexate, elevated hemoglobin or creatinine levels, and patient characteristics such as female sex [12].

In NHL, the incidence of thrombosis is approximately 6.5%, with high‐grade NHL showing a higher incidence (8.3%) compared to low‐grade NHL (6.3%) [11]. This is similar to the rate of VTE in our patients with LBCLs. For patients with Hodgkin lymphoma (HL), the incidence is lower, around 3.3%, with a higher incidence noted in advanced stages and those treated with dose‐dense regimens such as BEACOPP‐14 [13]. For patients undergoing allogeneic HSCT, the incidence of catheter‐related DVT and PE/ lower extremity DVT at 12 months was 4.2% and 4.8%, respectively. Hospitalization inpatient status was a strong predictor for the onset of these thrombotic events [14]. Therefore, the incidence of VTE observed in our CAR‐T treated population is similar to that seen in the general lymphoma population.

A retrospective, single‐center study of 148 patients with LBCL undergoing CAR‐T therapy reported an 11% incidence of new VTE events. Patients with Grade 3 or higher CRS, severe neurotoxicity, poor baseline performance status, and large tumor burden were identified as having a higher risk of developing VTE following CAR‐T therapy [15]. We observed similar findings in our study as well. Another meta‐analysis by Chitkara et al. [16], published in Current Oncology, reviewed Phase 2 and Phase 3 clinical trials and found a very low incidence of VTE, with only 3 out of 1017 patients experiencing VTE. Their study did not find a statistically significant association between increased VTE incidence and CRS or ICANS. There may be a variety of factors to account for the lack of consistency in our findings. Heterogeneity in the type of CAR‐T product (as this study used five different types of CAR‐T products as opposed to our study, which used one CAR‐T product), differences in thromboprophylaxis strategies, and the type of central venous access can be explored in future studies. A multi‐institutional study accounted for similar risk factors for VTE that we found in our study while creating a risk assessment model to predict VTE risk in lymphoma patients, including lymphoma histology and grade, in addition to therapy type, recent hospitalization, BMI, etc. [17].

Univariate analysis showed a significant relationship between tumor histology and VTE, as well as high‐grade (3 or 4 toxicity) ICANS and VTE. Our patients with PMBCL developed VTE almost five times more frequently than those with DLBCL and almost 10 times more frequently than those with TFL. Interestingly, all VTE events in the PMBCL cases were CVC‐related. Due to the small number of patients with PMBCL in our cohort, it is difficult to draw hypotheses on the reason for the association. Others have described that the anatomical and physiological phenotype of PMBCL, especially the presence of bulky mediastinal masses that can compress major vessels, such as the superior vena cava, could predispose patients to venous thrombosis as well as elevated levels of fibrinogen and D‐dimer [18]. In a study focusing on PMBCL, it was found that a significant proportion of patients (35.7%) developed VTE, including DVT and PE, compared to only 6.4% general incidence of thrombosis across all lymphoma types, which is consistent with our findings [18].

The association between high‐grade (3 or 4 toxicity) ICANS and VTE we observed is highly understudied with one current study documented by Schorr et al. [19]. Their proposed mechanism included elevated d‐dimer level, endothelial activation, and vascular injury, causing elevated serum markers such as von Willebrand factor and angiopoietin‐2, leading to a prothrombotic state [19, 20].

While our study demonstrates increased risk of VTE associated with tumor histology and high‐grade ICANS toxicity, our study does not allow us to determine if the complication of thrombosis is intrinsically related to CAR‐T cell therapy or to the type of patients who received it, or both. However, we can more closely examine and compare the incidence and risk factors of thrombosis in our cohort who received CAR‐T cell therapy to other study cohorts who received traditional chemotherapy regimens and/or CAR‐T therapy in future studies to be able to better prevent and address this somewhat common adverse event.

The timing of thrombosis development after starting CAR‐T therapy is also something we analyzed. Most thrombotic events took place within 4 weeks of CAR‐T therapy initiation in our cohort. This aligns with a median time of 23.5 days post‐infusion for thrombotic events from another single‐center retrospective study in adults with B‐cell malignancies and a median time of 29 days for thrombosis after CAR‐T therapy in patients with LBCL, or B‐cell ALL [19, 21]. A large meta‐analysis including 47 studies with a total of 7040 patients found that the incidence of VTE was highest within the first 6 months following CAR‐T therapy as well, with a pooled incidence of 2.4% per patient month (95% CI, 1.4%–3.4%). For follow‐up periods longer than 6 months, the incidence dropped significantly to 0.1% per patient‐month (95% CI, 0%–0.1%) [22]. These findings indicate that the risk of thrombosis is especially heightened in the initial month after CAR‐T cell therapy, highlighting the need for careful monitoring during this critical period and a potential study of the role of thromboprophylaxis strategies.

This study has several limitations. The relatively small sample size from a single institution reduces the generalizability of our findings. In addition, the lack of a control group receiving alternative therapies impedes direct comparison of VTE incidence between different treatment modalities. Moreover, the study period only captures short‐term VTE incidence within 6 months, potentially overlooking late thrombotic events. Further prospective, multicenter studies are warranted to validate these findings and explore the underlying mechanisms of VTE in CAR‐T therapy patients.

## 5 Conclusions

Our study demonstrates that there is a comparable risk for VTE in patients receiving CAR‐T cell therapy for a diagnosis of LBCL as in patients who have a diagnosis of lymphoma receiving chemotherapy. Interestingly, the incidence of VTE is higher in the first 4 weeks post CAR‐T, potentially due to the proximity of CAR‐T related toxicity. As these patients are constantly monitored during the first 4 weeks, and then only every 2–3 months after CAR‐T therapy, we may see these events of VTE early when patients are being monitored more closely. Further studies are needed to better understand the pathophysiology of VTE in patients who receive CART cell therapy, and the potential role of venous thromboprophylaxis within the first few weeks of CAR‐T.

## Author Contributions

**Neha Venkatesh**: conceptualization, writing – original draft, investigation. **Kevin Milligan**: data curation. **Julia Parrish**: data curation. **Yun Qing**: data analysis. **Ryan Sun**: data analysis. **Paolo Strati**: writing – review and editing. **Jeremy Ramdial**: writing – review and editing. **Amy Ayers**: writing – review and editing. **Sairah Ahmed**: writing – review and editing, supervision. **Cristhiam Rojas Hernandez**: conceptualization, writing – review and editing, supervision, data curation.

## Funding

Support grants for statistical analysis: R35GM154843 and U54CA302435.

## Conflicts of Interest

Paolo Strati is a consultant for Roche‐Genentech, AbbVie‐Genmab, Ipsen, Kite/Gilead, Hutchison MediPharma, AstraZeneca‐Acerta, ADC Therapeutics, Sobi, and TG Therapeutics; he has received research funds from Sobi, AstraZeneca‐Acerta, ALX Oncology, and ADC Therapeutics. Sairah Ahmed has research support to institution for clinical trials from Nektar, Merck, Xencor, Chimagen, Janssen, KITE and Genmab, has membership on Chimagen scientific advisory committee; she serves on Data Safety Monitoring Board for Myeloid Therapeutics; she is a consultant for ADC Therapeutics, KITE/Gilead. Cristhiam Rojas Hernandez has received research support from ANTHOS Therapeutics. The other authors declare no conflicts of interest.

## Data Availability

The data that support the findings of this study are available on request from the corresponding author. The data are not publicly available due to privacy or ethical restrictions. For original data, please contact CMRojas@mdanderson.org.
